# Using Digital Phenotypes to Identify Individuals With Alexithymia in Posttraumatic Stress Disorder: Cross-Sectional Study

**DOI:** 10.2196/83575

**Published:** 2025-11-13

**Authors:** Tomas Meaney, Vijay Yadav, Isaac Galatzer-Levy, Richard Bryant

**Affiliations:** 1 School of Psychology Faculty of Science University of New South Wales Sydney Australia; 2 Department of Psychiatry Grossman School of Medicine New York University New York City, NY United States

**Keywords:** mental health, digital health, alexithymia, digital phenotyping, machine learning, posttraumatic stress disorder, veterans

## Abstract

**Background:**

Alexithymia, defined as difficulty identifying and describing one’s emotions, has been identified as a transdiagnostic emotional process that impacts the course, severity, and treatment outcomes of psychiatric conditions such as posttraumatic stress disorder (PTSD). As such, alexithymia is an important process to accurately measure and identify in clinical contexts. However, research identifying the association between the experience of alexithymia and psychopathology has been limited by an overreliance on self-report scales, which have restricted use for measuring constructs that involve deficits in self-awareness, such as alexithymia. Hence, more suitable and effective methods of measuring and identifying those experiencing alexithymia in clinical samples are needed.

**Objective:**

In this cross-sectional study, we aimed to determine if facial, vocal, and language phenotypes extracted from 1-minute recordings of war veterans with PTSD describing a traumatic event could be used to identify those experiencing alexithymia.

**Methods:**

A total of 96 participants were included in this cross-sectional study. Specialized software was used to extract facial, vocal, and language features from the recordings. These features were then integrated into machine learning (extreme gradient boosting [XGBoost]) classification models that were trained and tested within a 5-fold nested cross-validation pipeline for their capacity to classify veterans scoring above the cutoff for alexithymia on the Toronto Alexithymia Scale-20.

**Results:**

The best performing XGBoost classification model trained in the nested cross-validation pipeline was able to classify those experiencing alexithymia with a good level of accuracy (average *F*_1_-score=0.78, SD 0.07; average area under the curve score=0.87, SD 0.12). Consistent with theoretical models and past research into phenotypes of alexithymia, language, vocal, and facial features all contributed to the accuracy of the XGBoost classification model.

**Conclusions:**

These findings indicate that facial, vocal, and language phenotypes incorporated in machine learning models could represent a promising alternative to identifying individuals with PTSD who are experiencing alexithymia. The further validation and use of this approach could facilitate more tailored and effective allocation of treatment resources to individuals experiencing alexithymia in clinical settings.

## Introduction

Alexithymia is defined as difficulty identifying and describing one’s own emotional states, in conjunction with externally focused attention [1,2]. In the attention-appraisal model of alexithymia, those with alexithymia are considered to have difficulties in attending to (due to their externally focused attention) and appraising (due to their difficulty identifying emotions) already occurring emotional responses to stimuli [3]. These issues with attending to and appraising emotional responses mean that it is difficult to subsequently describe them. The 3 core difficulties of people with alexithymia include internal orientation of attention, identifying and describing one’s emotional states, have been demonstrated to load directly onto the alexithymia construct and are consistent with the subscales of the primary alexithymia self-report measure, the Toronto Alexithymia Scale-20 (TAS-20) [4].

Alexithymia has been identified as a transdiagnostic risk factor for a range of psychiatric disorders [5-10]. A meta-analysis on the emotion processes relevant in schizophrenia found a large Hedge *g* effect size (1.05) for the association between alexithymia and schizophrenia [10]. A similarly large effect size has been found for the association between alexithymia and posttraumatic stress disorder (PTSD) [11]. Alexithymia has been conceptualized as an important mechanism in exacerbating PTSD symptoms and diminishing treatment response, given its strong association with emotional avoidance and its inhibition of the emotion processing required for gold-standard treatments, such as prolonged exposure therapy, to be effective [12]. This is consistent with findings that alexithymia following a traumatic event is predictive of the development of PTSD [13] and has a substantial influence on outcomes for PTSD interventions [14,15]. Accordingly, as with other transdiagnostic mechanisms of psychological distress, identifying alexithymia as it occurs in clinical settings, such that tailored treatment responses can be used, is important for ameliorating its impacts [12,16,17].

However, findings from most of these studies on the impacts of alexithymia are limited by their overreliance on self-report scales such as the TAS-20 [4]. This is problematic not only because self-report measures are prone to response biases [18,19] but also because alexithymia involves deficits in self-awareness that may impact the accuracy of self-report measures [20]. As such, there is a need for alternate and more construct-appropriate approaches to measure and identify alexithymia, particularly in clinical populations, in which it impacts symptom severity and treatment response.

The identification of alexithymia through individuals’ use of language is a construct-relevant approach that has been used in several studies. One such study of individuals with varying levels of alexithymia on the TAS-20 that analyzed their expressive writing samples using the Linguistic Inquiry and Word Count (LIWC) software (version 22; University of Texas) [21] found that those who scored higher on the TAS-20 used fewer words expressing affectivity, sadness, and future perspective [22]. Another study found that those scoring higher on the TAS-20 produced fewer emotion words and a less diverse range of emotion words yet did not have a general vocabulary deficit relative to low scorers [23]. A systematic review and meta-analysis of 29 empirical studies of language capacity in those with alexithymia found a modest association between language deficits (eg, emotion language use) and alexithymia [24], suggesting that language is not the only relevant expressive measure of alexithymia. This is consistent with findings that participants with alexithymia also demonstrated lower (or the same) physiological reactivity (heart rate, skin conductance, and facial electromyography) to negative stimuli, while reporting subjectively worse experiences than nonalexithymics [25-27]. Of particular relevance to PTSD, a distinction between high subjective distress and low arousal (heart rate) was found in the responses of those with alexithymia to fear imagery [27] and in the subjective report of emotional distress in individuals with PTSD who are alexithymic [28].

To enhance the measurement and identification of this multifaceted, clinically consequential construct of alexithymia, research could benefit from using facial, vocal, and linguistic features of emotional response. This approach is supported by a previous study showing that these features can be used in conjunction with machine learning (ML) models to identify those experiencing psychopathology following traumatic injury [29]. Facial, vocal, and linguistic features were extracted from recordings of participants’ responses to questions about their trauma. These features were integrated into an ML neural network model to predict provisional PTSD diagnoses made 1 month after the traumatic injury and variance in PTSD symptom severity. The models achieved an average accuracy score of 0.90 to classify PTSD, based on the contribution of linguistic, vocal, and facial features. As PTSD symptom severity has been associated with alexithymia in several studies [30,31], these features could be shared for individuals with PTSD who score above the cut-off for alexithymia. The possible consistency between the distinctive digital phenotypes of PTSD symptom severity and alexithymia is supported by the relevance of language sentiment and facial expressivity differences for distinguishing individuals with both alexithymia and PTSD [22,27].

This study aimed to estimate the capacity of an ML classification model, built with digital phenotype variables extracted from recordings of war veterans with probable PTSD (hereafter referred to as PTSD) in which they describe traumatic incidents they experienced, to accurately classify individuals with alexithymia. On the basis of the reviewed research, we hypothesized that veterans with PTSD with alexithymia could be classified with a good degree of estimated accuracy, which is what we found. We also hypothesized that language variables would be the most important variables for the estimated capacity of the classification model to classify individuals as alexithymic, given their association with alexithymia in past studies [22,23]. However, in line with the attention-appraisal model of alexithymia and past research demonstrating the different channels through which alexithymia can manifest, we hypothesized that vocal, facial, and linguistic variables would all contribute to the estimated capacity of the best-performing model to make classifications of alexithymia.

## Methods

### Participants

Participants for this study were 101 veterans of the Australian Defence Force who were recruited via the Trialfacts health research platform. Five participants were excluded due to missing questionnaire responses, leaving 96 participants. The inclusion criteria for this study were being a former member of the Australian Defence Force, having experienced a traumatic event, and scoring above 33 on the Posttraumatic Stress Disorder Checklist for *Diagnostic and Statistical Manual of Mental Disorders, Fifth Edition* (PCL-5). Most of the sample were men (78/96, 81%), and the mean age of participants was 52.38 (SD 11.80) years. The size of the sample was determined based on the requirements of the extreme gradient boosting (XGBoost) classification models to identify those scoring as alexithymic on the TAS-20. Our sample size was larger than those in previous studies examining language features distinctive of individuals high on alexithymia [22] and using an ML classification approach to identify individuals with PTSD [29].

### Stimuli

Before the experimental session, participants were asked to confirm if they felt comfortable discussing the traumatic event that had been affecting them the most. Participants were then asked to “think for a moment about a traumatic event you have been through” and to “tell me about this memory in detail... let yourself really try to get into this memory and how it made you feel.” Their responses were recorded for 1 minute.

### Measures

#### Posttraumatic Stress Disorder

The PCL-5 [32] is a 20-item self-report measure of the *Diagnostic and Statistical Manual of Mental Disorders, Fifth Edition*, symptoms of PTSD. It was used to determine if the veteran participants met criteria for probable PTSD, based on their self-report of PTSD symptoms related to traumatic events they experienced during their military service. Recruited individuals who served in the military were deemed to have probable PTSD if they scored above the cut-off of 33 on the PCL-5 [33]. The PCL-5 has been found to have high internal consistency (Cronbach α from 0.83 to 0.98) and convergent validity (correlations with other PTSD measures of value up to 0.93), indicating it has strong psychometric properties [33,34].

#### Depression

The Beck Depression Inventory-second edition (BDI-II) [35] is a 21-item self-report measure that was used to index the intensity of depression symptoms in the participants of this study. This measure was used to determine if differences between those who were alexithymic or not alexithymic could be explained by levels of depression, given the overlapping presentation features of alexithymia and depression [36,37]. The BDI-II has been shown to have strong convergent and criterion validity as well as high internal consistency (Cronbach α of 0.9) and reliability [38].

#### Digital Phenotypes

Participants’ recorded descriptions of their traumatic experiences were processed using the OpenWillis Python (Python Software Foundation) library [39] and LIWC-22 [21] software.

Facial indicators are based on the facial action coding system [40]. This coding system measures the intensity of activity in both individual and groups of muscles in the face (designated by particular facial action units) that have been found to relate to particular emotional experiences, such as the 6 primary emotions of happiness, sadness, surprise, fear, disgust, and anger. OpenWillis uses DeepFace to measure framewise intensity of facial action coding system units on a range of –1=expressivity of that emotion below baseline and 1=expressivity of that emotion above baseline to produce facial emotion expressivity scores. DeepFace has been found to have 97% accuracy in correctly identifying the facial landmarks of faces it has been previously trained on [41] and 94% accuracy in identifying human emotions [42]. OpenWillis also uses MediaPipe [43] to measure the frame-by-frame coordinates of 468 unique facial landmarks using its Face Mesh model. From this, it produces a measure of the mean frame-to-frame movement occurring at these coordinates across the length of the video (producing OpenWillis variables such as “Upper face expressivity”; see Multimedia Appendix 1 for a glossary of *OpenWillis* terms). MediaPipe was used as the building block for feature analysis in 1 study that was able to achieve 97% accuracy in correctly detecting human emotion [44].

OpenWillis uses Parselmouth to measure vocal variables [45]. Parselmouth is a Python implementation of the Praat software (University of Amsterdam) library [46]. Measured vocal variables include mean fundamental frequency, deviation in fundamental frequency, loudness, jitter, and shimmer of participants’ vocal production. Parselmouth also measures the percentage of frames without vocal content and the median duration of silences. In more recent updates, it has been able to examine more specific vocal features, such as cepstral peak prominence (CPP) and the mean, variance, and SD in mel-frequency cepstral coefficients. Praat software has been found to have good convergent validity (with other vocal software tools) and reliability in correctly identifying vocal features [47].

For language analysis, OpenWillis uses WhisperX to convert audio into text, which has a word error rate of 9.7%, outperforming previous speech-to-text models [48]. It uses the natural language processing Valence Aware Dictionary and Sentiment Reasoner (Massachusetts Institute of Technology) software [49] to analyze the extracted text in terms of language sentiment using a rule- or lexicon-based algorithm that produces mean scores from 1=negative sentiment to 1=positive sentiment. OpenWillis further measures the interaction between speech sentiment and first-person pronoun use (“first person language sentiment”). OpenWillis also uses LexicalDiversity [50], which is a natural language processing tool that measures lexical diversity in terms of moving average type-token ratio, which refers to the ratio of tokens (words) to the different types of words used in windows of 10 words at a time, that are then averaged across the whole segment of speech.

The LIWC library [51], which is the basis for the LIWC-22 software [21], is designed to process text files by counting the words in the text and calculating the percentage of words that correspond to each of the subdictionaries of LIWC (eg, the word “cry” would contribute to increasing the score of the subdictionaries of “emotion,” “affect,” and “verbs”). LIWC provides scores for each of its dictionaries, such as “power word use” and “word use related to feeling” [21]. Previous LIWC software has been found to have higher convergent and discriminant validity with other measures of emotion, such as self-report and rater coding, than competing text analysis software [52].

#### Affect Scale

Participants were asked to rate how they felt while describing the traumatic event on a 100-point verbal analogue scale (−100=means extremely negative, 0=neutral, and 100*=*extremely positive).

#### Alexithymia

The TAS-20 [4] was used to index the participant’s level of alexithymia. It has 20 items that each have a 5-point Likert rating scale (1=totally disagree and 5=totally agree) with items such as “I often don’t know why I am angry.” It has three factors: (1) difficulty describing feeling, (2) difficulty recognizing feeling, and (3) externally oriented thinking. The TAS-20 has an established cut-off score of 61 out of 100, over which an individual is deemed to have “alexithymia” [4]. A review of the TAS-20 measure determined that it has good factor validity, reliability, and internal consistency [53].

### Procedure

The study was conducted via Zoom for all participants because we recruited veterans from across Australia. Participants initially completed informed consent, then completed the PCL-5, the TAS-20, and a range of demographic measures via Qualtrics. Participants were informed that they would be asked to describe a traumatic event they had experienced in detail. These descriptions were audio-visually recorded using the Apple QuickTime app and the record function in Zoom (Zoom Communications, Inc). Each video recording had a frame rate of 60 frames per second. After providing the description, participants were asked to rate how they felt while talking about the traumatic event on the Analogue Affect Scale. After completing this process, they were debriefed on the nature and purpose of the study.

### Data Analysis

**Figure 1:**
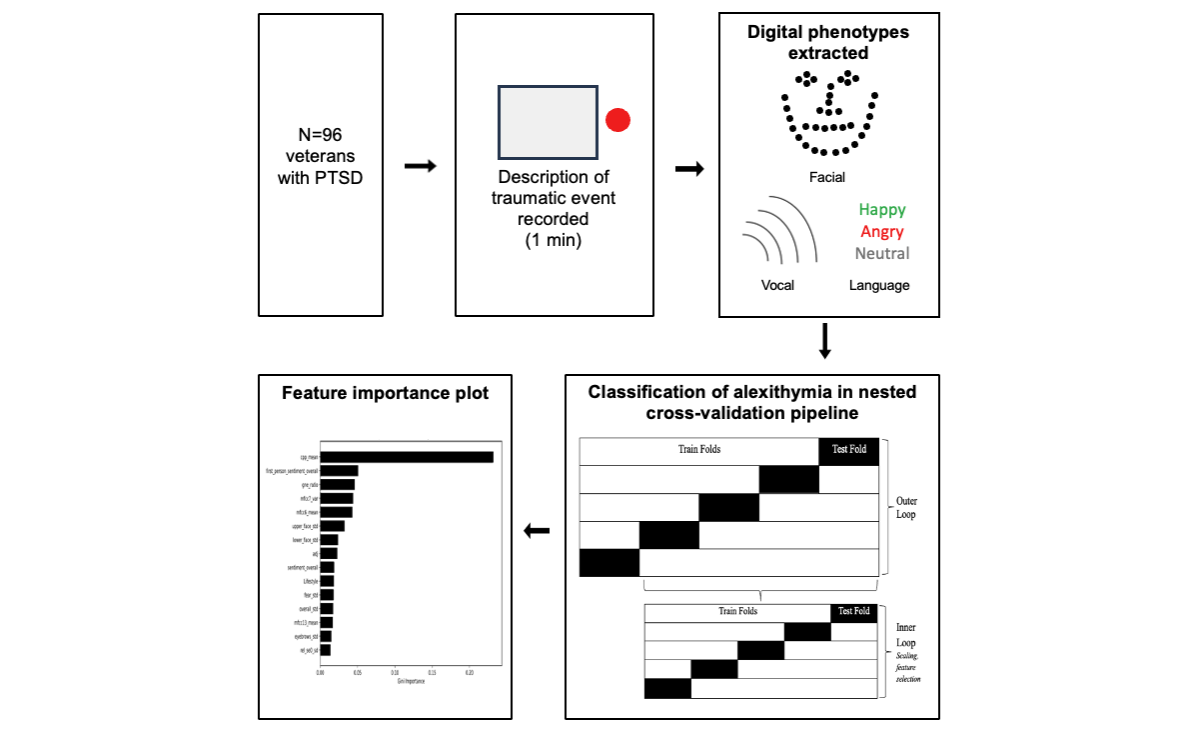
Experimental process. PTSD: posttraumatic stress disorder.

Participants were classified in terms of alexithymic status according to scores above or below the threshold of 61 on the TAS-20. This categorization resulted in 64 participants (n=53, 83% men; n=11, 17% women) being classified as alexithymic, and 32 participants (n=25, 78% men; n=7, 22% women) as nonalexithymic. XGBoost classification models were then built using the digital phenotype variables extracted from participant descriptions of traumatic events they have experienced. XGBoost classification models have been found to be both efficient and accurate in making classifications of features extracted from recordings of individuals with psychiatric conditions in past research [54]. In this study, these models were built using the scikit-learn package [55] in Python to attempt to classify individuals who scored in the “alexithymia” range on the TAS-20. The hyperparameters were set at the default values for the XGBoost classification algorithm in scikit-learn. Feature selection using the Recursive Feature Elimination method was completed within the inner folds in a 5-fold nested cross-validation pipeline to reduce the bias involved with doing feature selection on the whole sample and subsequently, the possibility of overfitting [56]. The number of features that contributed most to maximizing the precision score were selected and retained in a “best model.” The estimated classification performance of this model was evaluated across the outer 5 folds based on average scores for the precision, recall, *F*_1_-score, and area under the curve (AUC) metrics. Precision measures the rate at which the model is correctly identifying individuals as being in the “alexithymia” group by the number of times it is making the classification of “alexithymia.” Recall refers to the rate at which the model correctly identifies every individual with “alexithymia.” *F*_1_-scores are based on the harmonic mean of the precision and recall scores. The *F*_1_-score was used instead of the standard accuracy score in scikit-learn, as it has been found to be a more robust and subsequently appropriate measure when the two groups to be classified are imbalanced [57]. The AUC provides an indication of the probability that the model will rank each individual scoring above the cut-off for “alexithymia” as having a higher probability of being alexithymic than nonalexithymic. The average AUC scores derived from the receiver operating characteristic curve were used as they have been shown to be suitable for assessing classification with imbalanced datasets [58]. Variable feature importance was scored based on the average decrease in Gini impurity across all decision trees in the best-performing XGBoost model within the inner 5 folds of the nested cross-validation. The experimental process is represented in Figure 1.

### Ethical Considerations

All procedures for the study were approved by the University of New South Wales Human Research Ethics Committee (HC230175). All methods were performed in accordance with the relevant guidelines and regulations. Informed consent was obtained from all participants via an online form explaining what was involved in the study before signing. The privacy and confidentiality of participants’ data have been stringently maintained, with no identifying details or features being presented or included in the write up of the study. Participants were given a gift voucher of Aus $100 for participating in the study.

## Results

### Participant Characteristics

A chi-square test for participants’ sex at birth in the alexithymia and nonalexithymia groups found no significant group differences. One-way ANOVAs indicated no significant difference between those in the alexithymia and nonalexithymia groups for age, PCL-5, and BDI-II scores (Multimedia Appendix 1). This suggests that differences in digital phenotypes between those who were alexithymic and nonalexithymic were not driven by any of these examined covariates. Summary statistics for these variables are shown in Table 1.

**Table 1:** Participant characteristics.

Measures	Not alexithymic (n=32), mean (SD)	Alexithymic (n=64), mean (SD)
Age (y)	52.94 (11.83)	52.09 (11.91)
PCL-5^a^	48.38 (14.21)	48.05 (13.44)
BDI-II^b^	30.00 (11.66)	32.44 (9.96)

^a^PCL-5: Posttraumatic Stress Disorder Checklist for Diagnostic and Statistical Manual of Mental Disorders, Fifth Edition.

^b^BDI-II: Beck Depression Inventory-second edition.

### Nested Cross-Validation Results

**Figure 2:**
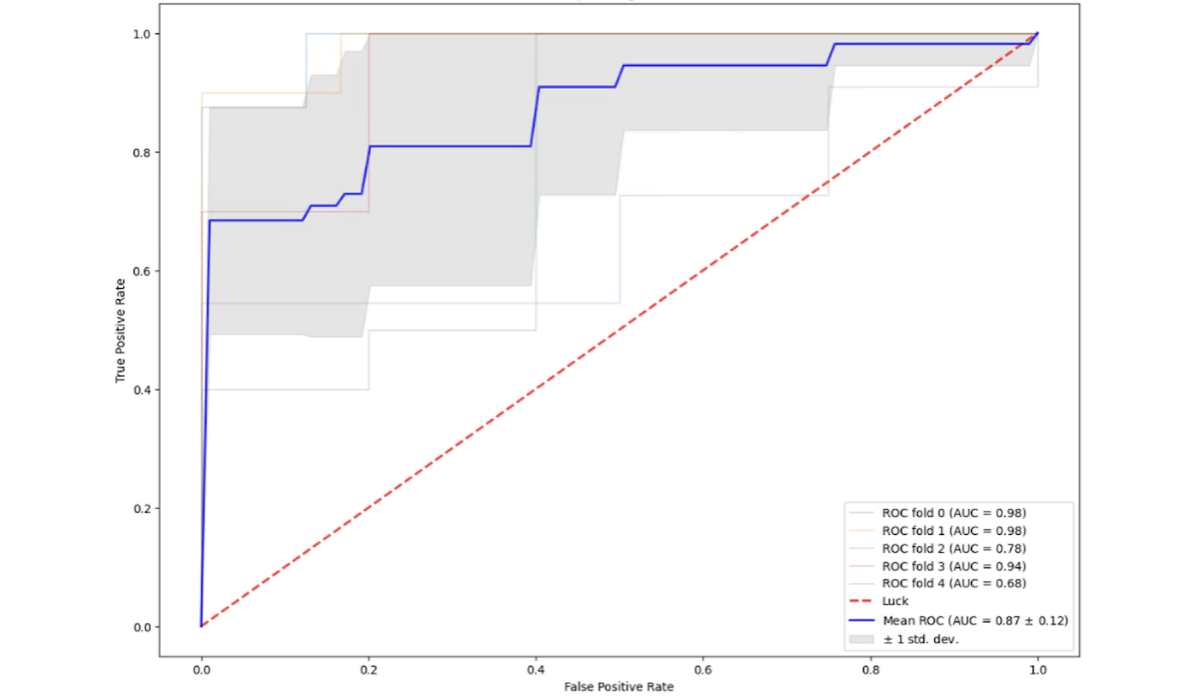
Receiver operating characteristic (ROC) curve, depicting the area under the curve (AUC) accuracy for alexithymia classification.

**Figure 3:**
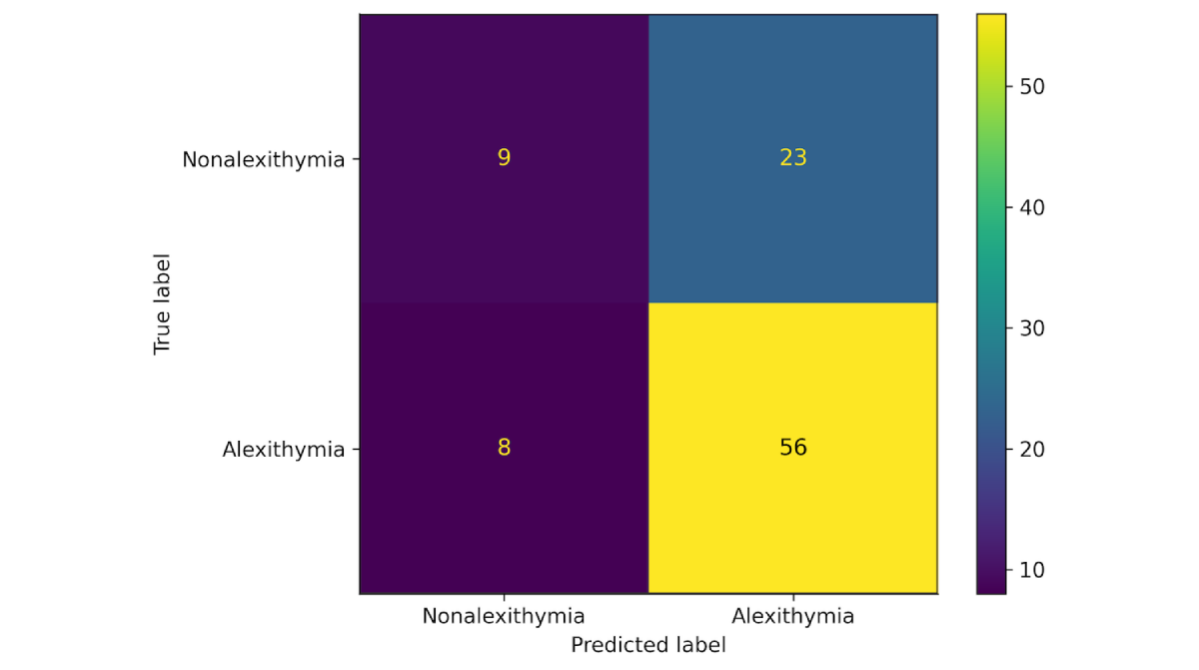
Confusion matrix for the classification of alexithymia or nonalexithymia by the extreme gradient boosting (XGBoost) model.

The best-performing XGBoost classification model for determining whether participants scored above the cut-off for alexithymia had an average precision of 0.71, an average recall of 0.87, an average *F*_1_-score of 0.78, and an average AUC of 0.87 (Figure 2) across the 5 outer testing folds, with a model that used 148 features (performance metrics across each fold are displayed in Table 2). The high recall score indicates that the XGBoost classification model was performing well at correctly identifying those individuals who scored above the threshold for alexithymia (“true positives”). However, in terms of the accuracy of all the alexithymia classifications it made, it was not performing as well, with 29% of those classifications being made incorrectly (“false positives”). These rates are illustrated in Figure 3, which is a confusion matrix depicting the predictions of the best performing classification model across the 5 outer folds relative to the true alexithymia labels. These predictions produced an average overall accuracy (*F*_1_-score) of 0.78 (SD 0.07). The average AUC of 0.87 (SD 0.12) suggests that the model was performing well at assigning a higher probability that an individual scoring above the cut-off for “alexithymia” was alexithymic across each of the outer folds. The receiver operating characteristic curve depicting AUC across the outer folds of the nested cross-validation pipeline for this model is displayed in Figure 3.

**Figure 4:**
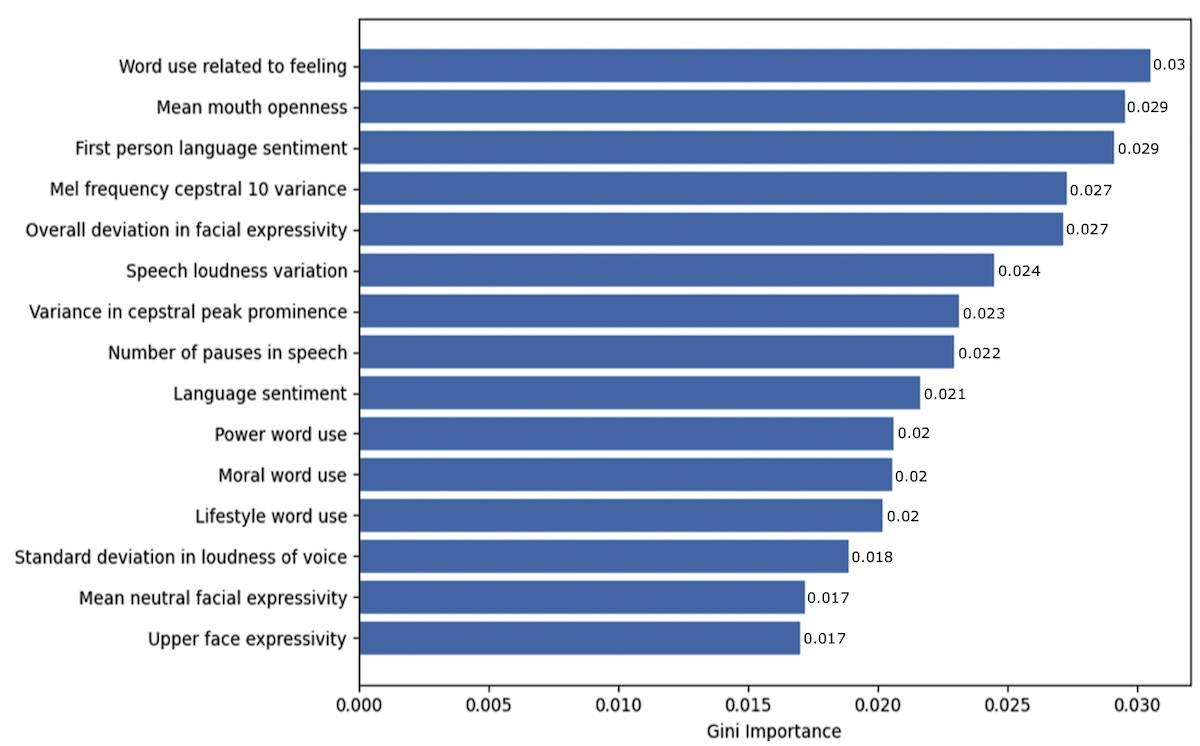
Feature importance plot depicting the most important features for the capacity of the extreme gradient boosting (XGBoost) model to accurately classify alexithymia.

Figure 4 displays the feature importance plot for the digital phenotype variables that were most important for classifying individuals as alexithymic based on their Gini importance scores. Language, facial, and vocal variables were important to the capacity of the XGBoost model to classify alexithymia, with “word use related to feeling” emerging as the predictor with the largest Gini importance score. Other language (such as “first person language sentiment” and “language sentiment”), facial (such as “mean mouth openness”), and vocal (eg, “mel frequency cepstral 10 variance”) variables were also important to the classification capacity of the XGBoost models. The importance of language, facial, and vocal variables highlights the value of taking this multimodal approach to identifying a construct such as alexithymia, which has a distinctive presentation across multiple domains.

**Table 2:** Performance metrics for the extreme gradient boosting classification model across the 5 outer folds of the nested cross-validation pipeline.^a^

Fold	Precision	Recall	*F*_1_-score accuracy
Fold 1	0.82	0.93	0.88
Fold 2	0.75	0.92	0.83
Fold 3	0.53	0.90	0.67
Fold 4	0.71	0.77	0.74
Fold 5	0.73	0.85	0.79

^a^The average across folds (SD) were as follows: precision, mean 0.71, SD 0.10; recall, mean 0.87, SD 0.06; *F*_1_-score accuracy, mean 0.78, SD 0.07.

## Discussion

### Principal Findings

This study examines the estimated capacity of an XGBoost classification model, built with digital phenotype variables extracted from recordings of war veterans with PTSD describing traumatic events they had experienced, to accurately classify those veterans with alexithymia. These models were built and evaluated in a nested cross-validation pipeline to minimize the impact of bias [56,59].

In line with our hypothesis, the XGBoost classification model tuned and built within the nested cross-validation pipeline demonstrated a level of accuracy and performance that indicated it could be used for classifying alexithymia in PTSD. Regarding the high recall score, with individuals who scored above the threshold for alexithymia, the model was estimated to be able to accurately classify these individuals as having alexithymia 87% of the time. The precision of the model, measuring how many of the classifications of alexithymia were accurate, was much lower, with only 71% of those classifications being accurate, suggesting the model may have been making too many “alexithymia” classifications. The XGBoost classification model had an average overall *F*_1_-score of 0.78, which is lower than the average *F*_1_-score achieved by the classification model for PTSD built using a similar approach [29]. This could be explained by this prior study attempting to classify trauma survivors with and without PTSD [29], whereas the present study focused only on identifying a subgroup of those with PTSD (those with alexithymia). The average AUC was 0.87, which is close to that identified for PTSD classification in a previous study and is considered model performance that suggests it has “considerable” clinical use [60]. However, this result needs to be interpreted with caution given its lack of stability, with the model only achieving an AUC of 0.55 in one of the folds. Overall, the performance of this XGBoost classification model suggests that such a model built with multiple digital phenotypes could be useful for identifying alexithymia in PTSD. This model must also be tested and validated on an independent sample of veterans with PTSD that was used in the model training process.

### Digital Phenotypes of Alexithymia in PTSD

This study was the first to examine multiple digital phenotypes in the context of alexithymia in PTSD, and in doing so found that not only language variables but also facial and vocal features were important for the estimated classification of alexithymia. Mean mouth openness was the most relevant facial feature that contributed to classification performance. This may show that differences in how much individuals spoke, as demonstrated by the openness of their mouths, were a factor in the accurate classification of alexithymia. The most relevant vocal feature was the mel-frequency cepstral coefficient variables (mel-frequency cepstral 10 variance) and variance in CPP. The importance of variance in CPP, which is a measure of voice pathology [61,62], to the estimated capacity of the XGBoost model to classify individuals as alexithymic is consistent with past findings linking alexithymia with experiences of voice pathology using other measures [63,64]. The contribution of these facial and vocal features to the estimated classification of alexithymia expands and enhances the understanding of the expressions of emotional experience that could be relevant to this construct.

As hypothesized, language variables were important to the estimated capacity of the XGBoost model to classify individuals as alexithymic. The language variables that had the highest Gini importance scores were associated with the use of feeling words, sentiment of language, and first-person pronoun use. This aligns with foundational theoretical understandings of alexithymia as a deficit in the description of experiences that are associated with feelings and emotional sentiment [3]. It also supports previous findings that those who score higher on the TAS-20 display differences in their expression of language sentiment [22,23]. In terms of first-person pronoun use, this aligns with theoretical understandings of alexithymia involving differences in the focus placed on oneself [2] and past findings that it is associated with differences in personal pronoun use [65]. This consistency between important predictors in an XGBoost classification model and expectations based on the research domain knowledge about that construct from theoretical models and past findings is an important indication of validity for ML models [66]. However, given that it was not only language variables alone that were important for the estimated classification of alexithymia but also vocal and facial variables, this aligns more closely with the attention-appraisal model’s understanding of alexithymia as a multifaceted construct [3] than that of the language hypothesis of alexithymia [67].

### Limitations

This study had several limitations. The size of the sample (N=96) is modest for an ML model such as XGBoost. The higher number of features (148 features) used in the best performing XGBoost classification model relative to the number of participants in the sample can increase the possibility of overfitting and deleteriously impact the stability of the model [68,69]. However, the use of nested cross-validation has been shown to minimize the impact of overfitting even in small samples, such that the models developed are replicated well in independent test sets [59,70]. Another limitation is that there was a large difference in the size of the groups to be classified (those scoring above or below the cut-off for “alexithymia” on the TAS-20), with more veterans in the sample scoring above the cut-off for “alexithymia.” This imbalance in groups impacts the capacity of the XGBoost classification model to be accurately evaluated. In the case of imbalanced classes, the classification of majority classes tends to be more accurate than that of the minority classes [71,72]. This phenomenon likely contributed to the much higher recall score found for this model relative to the precision score. However, this imbalance also reflects the generally higher incidence of alexithymia in veteran populations with PTSD, and trying to adjust these imbalances through cost-sensitive learning that incorporates oversampling or undersampling has been shown to have substantial limitations such as increasing overfitting [72-74]. The mostly male sample limits the generalizability of the digital phenotype findings to other PTSD populations (eg, civilians and women) and may have also contributed to the imbalance of groups on either side of the “alexithymia” cut-off, given that there is a small effect of sex on the TAS-20, with men generally scoring higher [75]. However, this higher proportion of men is representative of the defense force veteran sample used in this study [76]. Furthermore, this population is one in which alexithymia has a substantial impact on [77], which emphasizes the importance of improving our capacity to identify those who are alexithymic in this population.

### Conclusions

Overall, this study suggests that facial, vocal, and language indicators could be used in the identification of veterans with PTSD who are experiencing alexithymia. We emphasize that the model requires further validation in independent samples, but the findings represent an important first step and attest to the merits of continued research in this area. Particularly considering the limitations of self-report measures of alexithymia, this paradigm has the potential to advance research paradigms and the assessment of alexithymia in clinical settings. These advances could ultimately contribute to alexithymia being more easily identified in psychiatric contexts, leading to the allocation of more tailored and effective treatment resources for addressing the specific challenges associated with alexithymia. Future research involving the implementation of this approach in clinical settings is required to examine its feasibility, efficiency, and ease of integration into clinical assessment, as well as its accuracy in identifying alexithymia. The improved identification of alexithymia in PTSD would be an important step in ameliorating the specific impacts that alexithymia has on the course and treatment of psychiatric conditions such as PTSD [12].

## Appendix

Multimedia Appendix 1 Glossary of relevant digital phenotype variables.

## Abbreviations

AUC: area under the curve
BDI-II: Beck Depression Inventory-second edition
CPP: cepstral peak prominence
LIWC: Linguistic Inquiry and Word Count
ML: machine learning
PCL-5: Posttraumatic Stress Disorder Checklist for Diagnostic and Statistical Manual of Mental Disorders, Fifth Edition
PTSD: posttraumatic stress disorder
TAS-20: Toronto Alexithymia Scale-20
XGBoost: extreme gradient boosting

## Data Availability

The deidentified OpenWillis variable datasets analyzed during this study are available in the OSF repository [78]. The raw video recording data analyzed during this study are not publicly available due to privacy and ethical requirements.
