# Supplementary material for: Using Digital Phenotypes to Identify Individuals With Alexithymia in Posttraumatic Stress Disorder: Cross-Sectional Study
Source: JMIR Ment Health. 2025 Nov 13;12:e83575. doi: 10.2196/83575 (PMC12661231; doi:10.2196/83575)
Supplement: Multimedia Appendix 1 [file mental_v12i1e83575_app1.docx]

**Multimedia Appendix 1**

**Supplementary Table S1.** Glossary of relevant digital phenotype variables

| Variable | Definition |
| --- | --- |
| Overall deviation in facial expressivity | Standard deviation in the overall framewise displacement in facial landmark coordinates |
| Upper face expressivity | Mean framewise displacement in facial landmark coordinates in the upper half of the face |
| Mean neutral face expressivity | Standard deviation in the framewise displacement in facial landmark coordinates in the lower half of the face |
| Mean mouth openness | The mean ratio of mouth height by the minimum of the lower lip and upper lip height |
| Variance in cepstral peak prominence | Variance in cepstral peak prominence, which is a measure of dysphonia and breathiness |
| Standard deviation in loudness of voice | Variation in speech loudness as measured by the standard deviation of the energy of vocal segments longer than 100 milliseconds relative to its mean |
| Mel-frequency cepstral 10 variance | The 10^th^ Mel-Frequency Cepstral Coefficient (MFCC), which measures the short-term power spectrum of vocal production (mean and var(variance)) |
| Number of pauses in speech | Number of pauses (longer than 50 milliseconds and shorter than 2 seconds) in proportion to total speech |
| Language sentiment | Measure of overall valence of speech ranging from 0-1 |
| First person language sentiment | Percentage of first-person singular pronoun use multiplied by the score for overall sentiment of speech ranging 0-1 |
| Word use related to feeling | The use of words that relate to feeling |
| Power word use | The use of words related to power |
| Moral word use | The use of words related to morality |
| Lifestyle word use | The use of words used related to lifestyle |
